# Management of the patient with an acute coronary syndrome using oral anticoagulation

**DOI:** 10.1007/s12471-015-0727-0

**Published:** 2015-07-17

**Authors:** G.J.A. Vos, N. Bennaghmouch, K. Qaderdan, J.M. ten Berg

**Affiliations:** Department of Cardiology, St Antonius Hospital Nieuwegein, 2500, 3432 EM Nieuwegein, The Netherlands

**Keywords:** Oral anticoagulation, NOAC, Heparin, Bivalirudin, Prasugrel, Ticagrelor

## Abstract

A significant number of patients with atrial fibrillation, treated with oral anticoagulants, present with an acute coronary syndrome. Many of these patients have an indication for coronary angiography. The introduction of non-vitamin K antagonist oral anticoagulants (NOACs) and the novel P2Y12 inhibitors has generated new uncertainty about the optimal treatment regimen, whether triple or dual therapy should be given and which is the most beneficial P2Y12 inhibitor (clopidogrel, ticagrelor, prasugrel). In this article, we will summarise the practical advice on the management of acute coronary syndrome patients requiring oral anticoagulants following the recent consensus document of the European Society of Cardiology (ESC) Working Group on Thrombosis in association with the European Heart Rhythm Association (EHRA) and ESC guidelines.

## Introduction

Around 5–10 % of the patients presenting with an acute coronary syndrome (ACS) have atrial fibrillation (AF) and use oral anticoagulants (OAC) [1, 2]. In addition to OAC, these patients have an indication for dual antiplatelet therapy (DAPT), comprising a P2Y12 inhibitor (clopidogrel, prasugrel, ticagrelor) and aspirin. Triple therapy (OAC plus aspirin and a P2Y12 inhibitor) might thus be indicated; however, this may lead to an unacceptably high bleeding risk. To complicate clinical decision making further, the non-vitamin K antagonist oral anticoagulants (NOACs) have been introduced as an alternative to vitamin K antagonists (VKA) and are recommended in many patients because of their favourable risk profile and adequate stroke prevention [3, 4].

In this article, we will summarise the practical advice on the management of ACS patients requiring OAC, following the recent consensus document of the European Society of Cardiology (ESC) Working Group on Thrombosis in association with the European Heart Rhythm Association (EHRA)[5] and the ESC guidelines on ACS and atrial fibrillation [6–8].

## Antithrombotic management of a patient on an OAC in the cath lab

The majority of the patients presenting with ACS have an indication for coronary angiography (CAG). While the most commonly used anticoagulant, unfractionated heparin, reduces the risk of ischaemic complications during CAG and percutaneous coronary intervention (PCI), such as catheter thrombosis and stent thrombosis, it also increases the risk of bleeding [9]. When a patient is already on an OAC when going to the catheterisation laboratory (cath lab) we have to decide on: (1) whether to continue the OAC throughout CAG and PCI; (2) if the OAC treatment is interrupted, whether heparin bridging is needed and (3) which access site is optimal.

### Heparin bridging versus uninterrupted VKA

The AFCAS (prospective multicenter Atrial Fibrillation undergoing Coronary Artery Stenting) registry has shown that an uninterrupted approach with VKA was equally safe as bridging therapy during PCI while also being simpler and cost-effective [10]. Furthermore, addition of heparin to uninterrupted VKA during the procedure resulted in an increase of minor bleeding and access site complications (11.2 versus 5.5 %, *p* = 0.03) while not reducing thrombotic event rates. Also the recent substudy from the WOEST (What is the Optimal antiplatelet and anticoagulant therapy in patients with oral anticoagulation and coronary StenTing) trial has shown fewer early bleeding events following PCI in the uninterrupted VKA group and no difference in thrombotic events as compared with the patients undergoing bridging [11]. Although not based on randomised data, the ESC consensus document recommends the uninterrupted approach without bridging in patients on VKA [5].

### What to do if the patient is on an NOAC?

There are no randomised data whether to discontinue NOACs or proceed with CAG on treatment. The ESC consensus document states that for interventions with no clinically important bleeding risk, the procedure can be performed while the patient is being treated with an NOAC, as long as there is no peak concentration of the drug (thus 12–24 h after intake) [5]. For a minimal bleeding risk intervention, such as CAG, it is recommended to stop the NOAC 24 h before the procedure. In patients undergoing a procedure with a high bleeding risk (e.g. CABG) it is recommended to stop NOACs at least 48 h before the procedure. In patients treated with NOACs, bridging is usually not necessary, due to the fast-onset and offset action of these agents.

When there is no time to discontinue an NOAC, one has to remember that it provides insufficient anticoagulation during catheter intervention. An in vivo study by Yau et al. found that NOACs do not prevent contact activation such as occurs in a catheter [12]. A small randomised PCI trial by Vranckx et al., comparing pre-procedural dabigatran with standard procedural unfractionated heparin, also suggests that dabigatran does not provide sufficient anticoagulation as there was more need for bail-out anticoagulants and more periprocedural infarctions occurred in the dabigatran group [13]. The Dresden NOAC registry in patients undergoing PCI has shown that NOAC interruption without heparin bridging did not increase thrombotic event rates, while major bleeding complications were more common with heparin bridging [14]. Whether NOAC continuation and adding low-dose unfractionated heparin during CAG is a better option than NOAC discontinuation before CAG has to be proven before it can be recommended.

The use of fondaparinux in the cath lab is not an option because it also does not prevent catheter thrombosis in patients with ACS undergoing PCI as was shown by the OASIS trials, comparing fondaparinux with enoxaparin in patients presenting with non-ST-elevation ACS (OASIS V) [15] and ST-elevation myocardial infarction (OASIS VI) [16].

Multiple trials have shown that bivalirudin is an alternative to periprocedural heparin in ACS patients [17, 18]. The major advantage of bivalirudin is the reduced risk of bleeding as compared with heparin plus glycoprotein IIb/IIIa inhibitor (GPI). The ACUITY-PCI trial has shown that bivalirudin alone can safely replace heparin and GPI in ACS patients undergoing PCI with less major bleeding and with no differences in net clinical outcomes [19]. The BRIGHT trial, of which the preliminary results were presented during the Transcatheter Cardiovascular Therapeutics (TCT) 2014, confirms these findings. A reduction of 50–60 % in bleeding events in the bivalirudin-treated patients versus the heparin monotherapy or heparin+GPI groups (*p* = 0.041 and 0.001, respectively) was seen. However, the HEAT-PPCI trial has shown no difference in bleeding events but an increase in stent thrombosis with bivalirudin, compared with heparin, when bailout GPI were used in both arms [20].

In summary: according to the ESC consensus document NOACs should be stopped 24 h before CAG and UFH bolus (a dosage of 60 IU/kg may be considered) or bivalirudin (high bleeding risk) should be used during the procedure [5]. When there is no time to discontinue the NOAC (ongoing ischaemia) additional heparin or bivalirudin should be used.

### Choice of approach

The radial approach is preferred to the femoral approach as it leads to less bleeding and vascular complications [21]. In the RIVAL (Radial versus Femoral Access for Coronary Intervention) trial (*n* = 7021), the primary composite endpoint, death, MI, stroke or bleeding at 30 days, did not differ between the femoral and radial access sites; however major vascular complications were lower in the radial group (1.4 vs. 3.7 %, *p* < 0.0001), which was primarily due to fewer haematomas [22]. In a study by Baker et al. (*n* = 255) in patients on OAC undergoing PCI, the radial approach was associated with a decrease in any vascular or bleeding complication compared with the femoral approach (1.6 vs 8.1 %, *p* = 0.02) [23].

**Table:** 

**Summary**
The uninterrupted OAC without bridging approach is recommended in ACS patients on VKA [10, 11] (Level of Evidence: B)
It is recommended to stop NOACs in all patients 24 h before coronary angiography, bridging is usually not needed [3, 5] (Level of Evidence: C)
Standalone NOAC provides insufficient anticoagulation during catheterisation and therefore either heparin or bivalirudin (in ACS patients) should be used [9, 10] (Level of Evidence: C)
When a parenteral anticoagulant is needed to support PCI in a patient at high risk of bleeding, bivalirudin should be considered as an alternative to unfractionated heparin [17, 18] ^a^(Level of Evidence: A)
Radial access is the default choice for coronary angiography/intervention to minimise the risk of access site-related bleeding depending on the operator in all patients [15, 17] (Level of Evidence: A)
New-generation DES (or BMS) are preferred over first-generation DES (Level of Evidence: C)

^a^Increased risk of stent thrombosis, the HEAT-PPCI trial did not show a significant difference between bivalirudin and heparin, Note: Levels of evidence following the European Society of Hypertension (EHS)/ESC guidelines classification scheme.

Level of Evidence A: Data derived from multiple randomised clinical trials or meta-analyses.

Level of Evidence B: Data derived from a single randomised trial, or nonrandomised studies.

Level of Evidence C: Only consensus opinion of experts, case studies, or standard-of-care.

### Antithrombotic management of the first year after PCI

Most thromboembolic events in AF are due to systemic embolism of thrombus from the left atrial appendage. The pathophysiological mechanism for thromboembolic events is fibrin mediated, whereas the most important underlying pathophysiological mechanism in ACS is platelet aggregation with secondary activation of the coagulation cascade [24]. In line with the pathophysiological mechanisms, the ACTIVE W trial has shown that VKA is the superior treatment for AF patients as compared with DAPT, while for patients undergoing stenting, DAPT was superior to the combination of warfarin and aspirin in the prevention of stent thrombosis [25]. AF patients with an indication for OAC should therefore continue to use OAC in combination with one or more antiplatelet agents when presenting with ACS. The goal is to maintain a reduced INR of 2.0–2.5 during combination of VKA and antiplatelet therapy.

First-generation drug-eluting stents (DES) require a longer period of DAPT than bare-metal stents (BMS) to prevent stent thrombosis, since observational studies have shown an increase in the occurrence of late and very late stent thrombosis. The second-generation DES have theoretical advantages over the first generation, such as a thin-strut design, reduced polymer layer, as well as novel anti-prolific drugs. Recent evidence from multiple clinical trials testing a short duration (3–6 months) versus 12–24 months of DAPT therapy has shown no significant difference in thrombotic endpoints, while the risk of bleeding was reduced with a shorter DAPT duration. [26–29] In contrast, the large DAPT Study has shown that DAPT for 30 months versus 12 months after DES reduced the risk of stent thrombosis and major adverse cardiac and coronary events (MACCE). While bleeding was more common in subjects continuing DAPT treatment, major or fatal bleeding were uncommon and not statistically significant [30]. In summary, the above trials suggest that especially in patients requiring a combination of OAC+P2Y12 inhibitor after second-generation DES implantation, a shorter treatment duration could be considered beneficial due to less risk of bleeding events.

### Is triple therapy needed in all these patients?

On theoretical grounds, triple therapy (OAC+aspirin+P2Y12 inhibitor) seems to be the most effective treatment in the prevention of thrombotic events in patients with AF presenting with ACS; this applies to both conservatively treated patients and those undergoing PCI with stent implantation. However, large prospective studies have shown that triple therapy is associated with a significant (frequently unacceptable) increase (2–44x) in major bleeding. ([31–33; Table 1.)

**Table 1:** Triple therapy versus dual therapy (OAC+aspirin or P2Y12 inhibitor)

	Sarafoff et al. (registry) [31]	Lamberts et al. (registry) [32]	AFCAS (registry) [33]	WOEST (Multicentre, RCT) [34]
Population	Indication OAC+DES	AF+ACS and/or PCI with stenting	AF+PCI with stenting	OAC indication+PCI with stenting
No. of patients	515 (209 DAPT, 306 TT)	11480 (Danish national registry)	975 (914 included in final analysis)	563
Follow-up in months	24	12	12	12
Therapy	Triple vs double therapy	TT vs vitamin K antagonist+aspirin or clopidogrel	TT vs double therapy (OAC+clopidogrel)	TT vs dual therapy
**Ischaemic endpoint**	Death, MI, ST or stroke	Cardiovascular death, MI, or stroke	Major adverse cardiac/cerebrovascular events	Death, myocardial infarction, stroke, target-vessel revascularisation, and stent thrombosis
Event rate, %	14.1 vs 18.0	14.2 vs 9.7	22.0 vs 18	17.6 vs 11.1
Hazard ratio (95 % CI)	0.76 (0.48–1.21)	HR 1.15; 95 % CI 0.95–1.40	NR (93.9 vs 94.5 % event free)	0.60 (0.38–0.94])
*P*-value	0.25	-	0.72	0.027
**Bleeding endpoint**	TIMI major	Hospitalisation for fatal or nonfatal bleeding	BARC major bleeding	TIMI major
Event rate, %	1.4 vs 3.1	14.2 vs 9.7	10 vs 7	5.6 vs 3.2
Hazard ratio (95 % CI)	NR	HR 1.41 (1.10–1.81)	NR (93.7 vs 95.8 event free)	0.56 (0.25–1.27)
*P*-value	NR	NR	0.43	0.159

*ACS* acute coronary syndrome, *AF* atrial fibrillation, *BARC* Bleeding Academic Research Consortium, *CI* confidence interval, *DAPT* dual antiplatelet therapy, *HR* hazard ratio, *MI* myocardial infarction, *NR* not reported, *OAC* oral anticoagulants, *PCI* percutaneous coronary intervention, *RCT* randomised controlled trial, *ST* stent thrombosis, *TIMI* Thrombolysis in Myocardial Infarction, *TT* triple therapy.

The randomised WOEST trial demonstrated that dual therapy significantly reduced bleeding events and MACCE in patients with AF undergoing PCI, when compared with triple therapy [34]. The study was, however, small and not powered to show a difference in thrombotic events such as stent thrombosis. Nevertheless, support for the results from the WOEST trial comes from multiple large registries [31–33]. In a nationwide Danish registry of antithrombotic use in AF patients discharged after myocardial infarction and PCI (*n* = 11,480), dual therapy (OAC+clopidogrel) reduced bleeding events (HR 0.78; CI 0.55–1.12) and thrombotic events (HR 0.69; CI 0.48–1.00) when compared with triple therapy [32]. An analysis of the AFCAS registry has shown that one-year efficacy and safety of all strategies (triple therapy, DAPT and dual therapy) in patients with AF undergoing coronary artery stenting were comparable after propensity matching, suggesting that dual therapy is as safe as triple therapy in regard to thrombotic risk [33].

### Can noacs be combined with antiplatelet therapy?

The evidence concerning the combination of NOACs and antiplatelet therapy is scarce. However, since most phase III clinical trials in patients with AF demonstrated that NOACs, in general, reduced bleeding events without compromising efficacy, they might be a better alternative than VKA in these patients presenting with ACS. This may especially be the case when a patient has a low time-in-therapeutic-range on VKA. However, even low-dose (2.5–5 mg) rivaroxaban (lower than the therapeutic dose [20 mg] for AF), added to DAPT in patients presenting with ACS, was associated with an increased risk of major bleeding including intracranial haemorrhage in the randomised ATLAS ACS 2– TIMI 51 trial [35]. As the occurrence of especially myocardial infarction was reduced by adding rivaroxaban, this trial lends support that NOACs may reduce ischaemic events after ACS.

A meta-analysis by Oldgren et al. [36] of all phase II and III NOAC trials investigating the combination of an NOAC with single (aspirin) or dual (aspirin and clopidogrel) antiplatelet therapy in the ACS setting has shown a modest reduction in cardiovascular events with a substantial increase in bleeding, most pronounced in patients using DAPT. To make this issue even more complicated: in the above trials, clopidogrel was used while the standard of care in ACS nowadays includes the more potent novel P2Y12 inhibitors (ticagrelor or prasugrel).

In patients already using an NOAC before PCI, the ESC consensus document recommends to continue the use of the NOAC and combine it with antiplatelet therapy. In addition, it is advised that when NOACs are used in dual or triple therapy, to consider the use of the lower dose tested for stroke prevention in AF (dabigatran 110 mg twice daily, rivaroxaban 15 mg once daily or apixaban 2.5 mg twice daily) [5].

The advised duration of triple therapy in ACS patients ranges from 4 weeks for patients with a high bleeding risk (HAS-BLED ≥ 3) to 6 months for patients with a low to moderate bleeding risk (HAS-BLED 0–2), followed by dual therapy (clopidogrel 75 mg/day (or alternatively, aspirin 75–100 mg/day).

### New-generation P2Y12 inhibitors

While the newer generation of platelet inhibitors (ticagrelor or prasugrel) have been introduced to provide stronger platelet inhibition and have shown to be more effective in reducing death, MI and stroke as compared with clopidogrel, they have a higher bleeding risk [37, 38]. Data on the use of new-generation P2Y12 inhibitor in the context of dual or triple therapy is still limited. A recent small observational study (*n* = 355) by Sarafoff et al. has shown that prasugrel as part of triple therapy in patients on VKA increased thrombolysis in myocardial infarction (TIMI) minor and major bleeding events (28.6 versus 6.7 %; adjusted HR 3.2, 95 % CI 1.1–9.1), while not significantly reducing thrombotic adverse [31]. Another small (*n* = 255) observational study by Braun, comparing patients on dual therapy with ticagrelor versus a historical control cohort discharged with triple therapy, thrombotic and bleeding events were similar [39].

Until more evidence becomes available, the use of ticagrelor or prasugrel in the context of double or triple therapy is not recommended as stated in the ESC consensus document. The PIONEER-AF and RE-DUAL trials will evaluate the safety of two different rivaroxaban and dabigatran treatment strategies as compared with VKA utilising various combinations of antiplatelet therapy using clopidogrel or prasugrel/ticagrelor in patients with non-valvular AF who undergo PCI with stent placement (NCT01830543, NCT02164864).

**Table:** 

**Summary**
Triple therapy is associated with an increased risk of major bleeding. Depending on bleeding risk and indication (stable coronary artery disease or ACS), triple therapy should be given for a short duration (1–6 months) followed by dual therapy (clopidogrel 75 mg/day (or alternatively, aspirin 75–100 mg/day) [6] (Level of Evidence: B)
Novel P2Y12 receptor inhibitors (prasugrel and ticagrelor) should not routinely be part of a triple therapy regimen (only in select cases such as stent thrombosis) (Level of Evidence: C)

## Long-term management (stable cardiovascular disease)

In patients with stable cardiovascular disease (i.e. ACS with or without stent placement ≥ 1 year ago), the risk of stent thrombosis and/or recurrent of spontaneous coronary, is diminished. Therefore OAC monotherapy (VKA or NOAC) should be used, since OAC+aspirin is associated with excess bleeding and does not reduce thromboembolic events. However, evidence for the safety of NOAC monotherapy is indirect. Addition of clopidogrel 75 mg daily, or as an alternative, aspirin 80 mg/day should only be considered in patients with a very high thrombotic risk.

**Table:** 

**Summary**
In a patient with AF and stable vascular disease (free of any acute ischaemic events or repeat revascularisation for > 1 year) the patient should be managed with OAC alone (whether NOAC or a VKA). (Level of Evidence: B)

## Conclusion

In recent years more data on the management of patients with OAC and ACS has become available. However, most evidence is still primarily based on observational studies. The introduction of NOACs and the novel P2Y12 inhibitors has generated new uncertainty on the optimal treatment regimen, whether triple therapy or dual therapy should be given and which is the most beneficial P2Y12 inhibitor (clopidogrel, ticagrelor, prasugrel). There is no strong evidence to suggest that NOACs behave differently to VKAs in the setting of ACS. While evidence in many areas is still limited, new trials will clarify the risks and benefits of different antithrombotic and antiplatelet combinations and the optimal duration of therapy.

### Funding

None.

### Conflict of interest

Dr. ten Berg has received speaker’s fees from AstraZeneca, Merck, and Lilly; and has received a research grant from AstraZeneca. All other authors have reported that they have no relationships relevant to the contents of this paper to disclose.
